# Response of cotton phenology to climate change on the North China Plain from 1981 to 2012

**DOI:** 10.1038/s41598-017-07056-4

**Published:** 2017-07-26

**Authors:** Zhanbiao Wang, Jing Chen, Fangfang Xing, Yingchun Han, Fu Chen, Lifeng Zhang, Yabing Li, Cundong Li

**Affiliations:** 1 Institute of Cotton Research of the Chinese Academy of Agricultural Sciences, State Key Laboratory of Cotton Biology, Anyang, Henan 455000 China; 2 College of Agronomy, Agricultural University of Hebei/Hebei Key Laboratory of Crop Growth Regulation, Baoding, 071001 China; 3 College of Agronomy and Biotechnology, China Agricultural University Key Laboratory of Farming System, Ministry of Agriculture, China and, Beijing, 100193 China

## Abstract

To identify countermeasures for the impacts of climate change on crop production, exploring the changes in crop phenology and their relationship to climate change is required. This study was based on cotton phenology and climate data collected from 13 agro-meteorological experimental stations and 13 meteorological stations on the North China Plain from 1981 to 2012. Spatiotemporal trends in the cotton phenology data, lengths of the different growing phases, mean temperatures, and rainfall were analyzed. These results indicated that warming accelerated cotton growth, advanced cotton phenology, and shortened the growing period of cotton. However, harvest dates were significantly delayed at 8 (61.5%) stations, the length of both the flowering-boll opening and boll opening-harvest periods increased at 10 (77.0%) stations, and a positive correlation was found between the mean temperature and the length of the whole growing period at 10 (77.0%) stations. Therefore, cotton practices and cultivars on the North China Plain should be adjusted accordingly. The response of cotton phenology to climate change, as shown here, can further guide the development of options for the adaptation of cotton production in the near future.

## Introduction

Climate change is projected to dramatically affect crop production across broad regions of the world in the 21^st^ century^1^. Many recent studies have shown that climate change has a significant impact on crop production^2–4^. Lobell *et al*.^2^ reported that each degree day above 30 °C decreased the African maize yield by 1% under optimal precipitation conditions and by 1.7% under drought conditions. Moreover, Liu *et al*.^3^ showed that over the past 40 years, increased temperatures have caused the northward expansion of the northern limit of maize in Northeast China. Chen *et al*.^4^ studied the impact of climate change on cotton yields in China, and the results showed that climate change decreased cotton yields. However, beneficial effects were found in the cotton-growing regions of Northwest China from 1961 to 2010. Therefore, the potential impact of climate change on the development and productivity of field crops is of great concern and has been evaluated extensively through simulation models, statistical analyses, and field experiments^5–7^.

Phenology refers to periodic life-cycle events and is important for plant survival and reproduction^8^. Plant phenology is strongly controlled by short- and long-term climate variability; consequently, phenological shifts have been among the strongest biological indicators of climate change^9, 10^. Phenological studies significantly contributed the conclusions of the International Panel of Climate Change’s (IPCC’s) Fourth Assessment Report that “there is very high confidence of biological responses to climate change, based on more evidences from a wider range of species”^11^. Therefore, investigations of the spatiotemporal changes in crop phenology and the relationships between phenology and climate change are important for understanding the processes and mechanisms underlying crop responses and adaptations to agro-meteorological stressors and ongoing climate change^12, 13^.

In this context, a number of studies on crop phenology based on observations of phenological events^14–16^, satellite analyses^17, 18^, and climatological analyses^3, 19^ have been reported. Tao *et al*.^15^ found that increased temperatures advanced maize heading and maturity dates and reduced the duration of the whole growing period at 81.3%, 82.1% and 83.9% of the examined stations in China by 3.2, 6.0 and 3.5 days per decade on average, respectively, from 1981 to 2009. Lu *et al*.^17^ developed a curve-fitting approach to enhance phenological extraction for winter wheat from a time series of SPOT-VEGETATION products. Using observed climatic and maize phenological data collected from 1990 to 2012. Li *et al*.^12^ analyzed the correlations between maize phenology and temperature in Northeast China based on data collected from 1990 to 2012. Indeed, most studies have focused on principal food crops, and research related to the impact of climate change on cotton phenology therefore remains scarce.

The North China Plain is one of the most important cotton production regions in China, encompassing 19,788 km^2^ and accounting for 38.66% of the area sown with cotton in China from 2005 to 2015^20^. The average surface temperature of this region has increased at a rate of 0.25 °C per decade over the past 50 years^21^. These large increases in temperature affect cotton growth and threaten the stability of cotton production in China. Therefore, investigations examining the effects of climate change on cotton phenology on the North China Plain are urgently needed. Furthermore, to understand the processes and mechanisms controlling the response of cotton to ongoing climate change, changes in cotton phenology that have occurred during the past several decades on the North China Plain in response to climate change must be investigated.

The objectives of this study were (1) to investigate the spatiotemporal changes in cotton phenology on the North China Plain, (2) to examine the relationships between climate change and the lengths of different cotton growing periods, and (3) to understand the consequences of phenological changes and their implications for cotton production and adaptation to climate change on the North China Plain.

## Results

### Shifts in the timing of cotton phenophases

**Figure 1:**
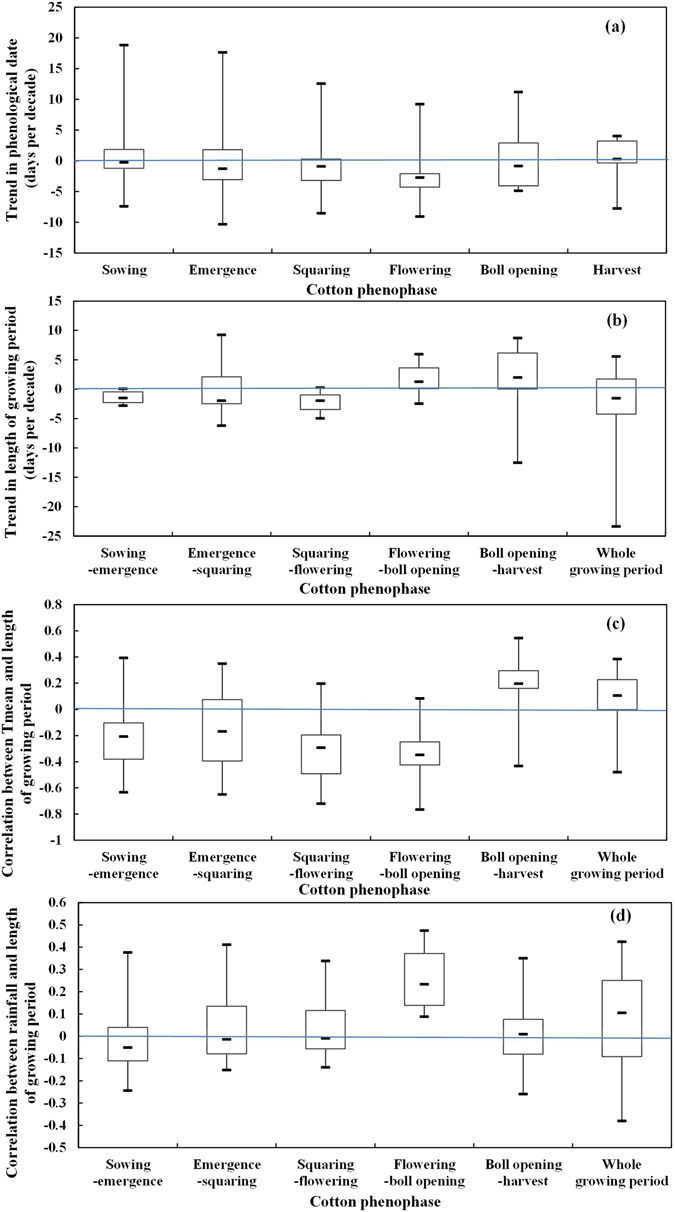
Trends in phenological dates (**a**) and the length the of growing period (**b**), correlations between Tmean and the length of the growing period (**c**), and correlations between rainfall and the length of growing period (**d**). The blue line is the zero line. Trends above or below the line represent a delay or advance, respectively, in phenology (**a**) or a prolonged or shortened growing period (**b**). The correlation coefficients above or below the line represent a positive or negative correlation (**c,d**).

**Figure 2:**
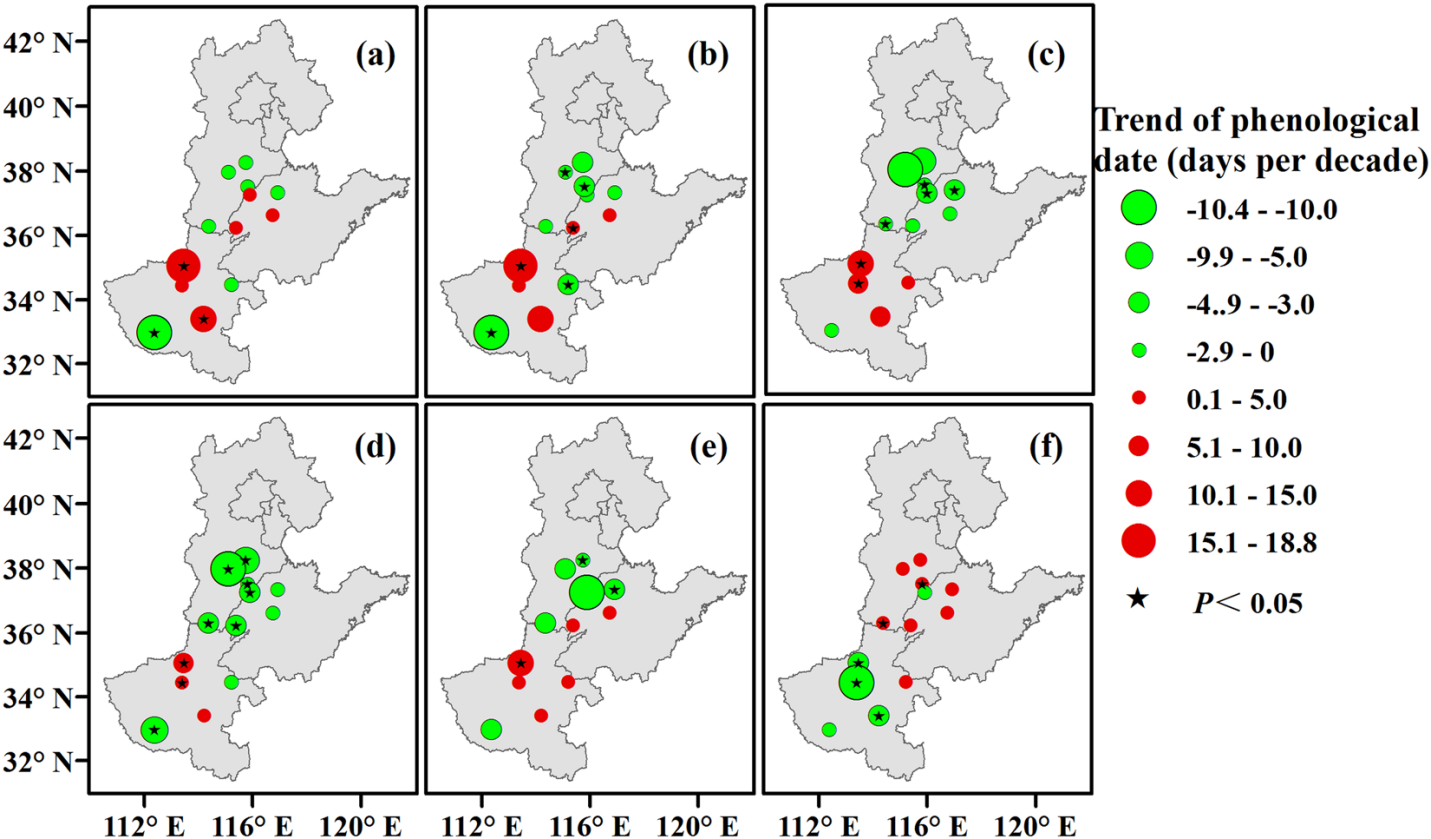
Spatiotemporal changes in the cotton sowing (**a**), emergence (**b**), squaring (**c**), flowering (**d**), boll opening (**e**), and harvest (**f**) dates across the North China Plain from 1981 to 2012. Green and red circles represent the trends of the phenological date. Black pentagrams represent the trends at the stations, with a significance level of 0.05. Maps were generated using ArcGIS 10.1 (ESRI Inc, Redlands, CA, USA, http://www.esri.com/).

The sowing, emergence, squaring, flowering, and boll opening dates of cotton were advanced by 0.24, 1.29, 0.91, 2.71 and 0.82 days per decade on average, respectively, whereas the cotton harvest date was delayed by 0.28 days per decade on average from 1981 to 2012 (Fig. 1a). Among the 13 stations, sowing, emergence, squaring, flowering, and boll opening dates were advanced at 7 (53.9%), 8 (61.5%), 9 (69.2%), 10 (76.9%), and 7 (53.8.1%) stations, respectively, and 8 (61.5%) stations presented delayed harvest dates (Fig. 2). Furthermore, from 1981 to 2012, the cotton flowering date was significantly advanced at 7 (53.9%) of the 13 stations, mainly in Hebei and Shandong Provinces, whereas the date was significantly delayed at 2 (15.4%) stations, mainly in Henan Province.

### Shifts in the length of cotton phenophases

**Figure 3:**
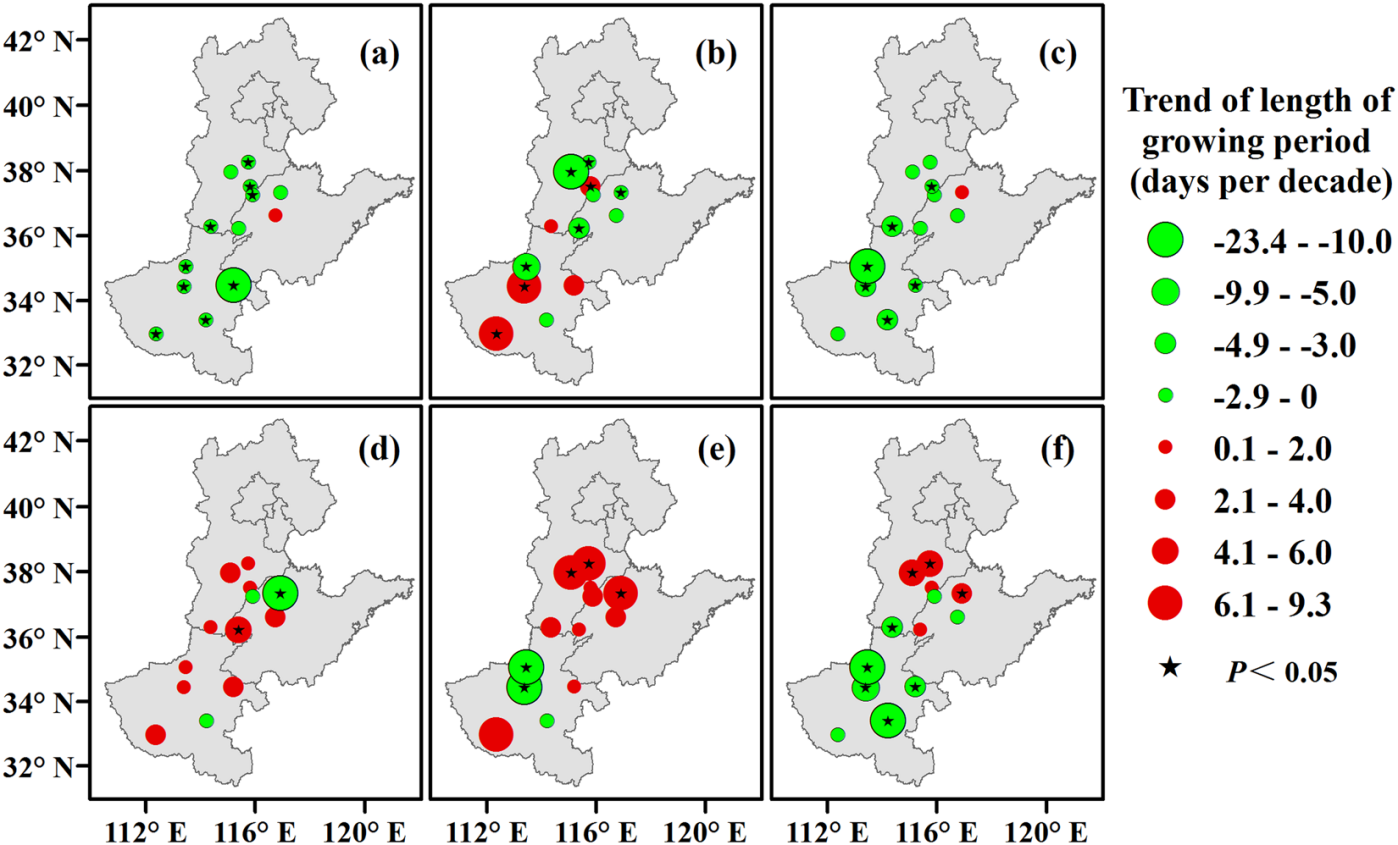
Spatiotemporal changes in the lengths of the sowing-emergence (**a**), emergence-squaring (**b**), squaring-flowering (**c**), flowering-boll opening (**d**), and boll opening-harvest (**e**) periods and the whole growing period (**f**) of cotton across the North China Plain from 1981 to 2012. Green and red circles represent the trends of the lengths of phenological periods. Black pentagrams represent the trends at the stations, with a significance level of 0.05. Maps were generated using ArcGIS 10.1 (ESRI Inc, Redlands, CA, USA, http://www.esri.com/).

In general, the sowing-emergence, emergence-squaring, and squaring-flowering periods and the whole growing period of cotton were shorter by 1.50, 1.94, and 1.96 days per decade on average, respectively, while the flowering-boll opening and boll opening-harvest periods were longer by 1.28 and 2.20 days per decade on average, respectively, (Fig. 1b). The lengths of the sowing-emergence, emergence-squaring, and squaring-flowering periods and the whole growing period decreased at 12 (92.3%), 8 (61.5%), 12 (92.3%) and 8 (61.5%) stations, respectively, and this decrease was significant at 9 (69.2%), 5 (38.5%), 6 (46.2%), and 5 (38.5%) of these stations, respectively, among the 13 North China Plain stations (Fig. 3). In contrast, the lengths of both the flowering-boll opening and boll opening-harvest periods increased at 10 (77.0%) stations, with 1 (7.7%) and 3 (23.1%) of the 13 North China Plain stations showing significant increases during these respective periods. Furthermore, while the length of the whole growing period increased at the northern North China Plain stations, it decreased at most of the stations.

### Relationship between cotton phenology and Tmean

**Figure 4:**
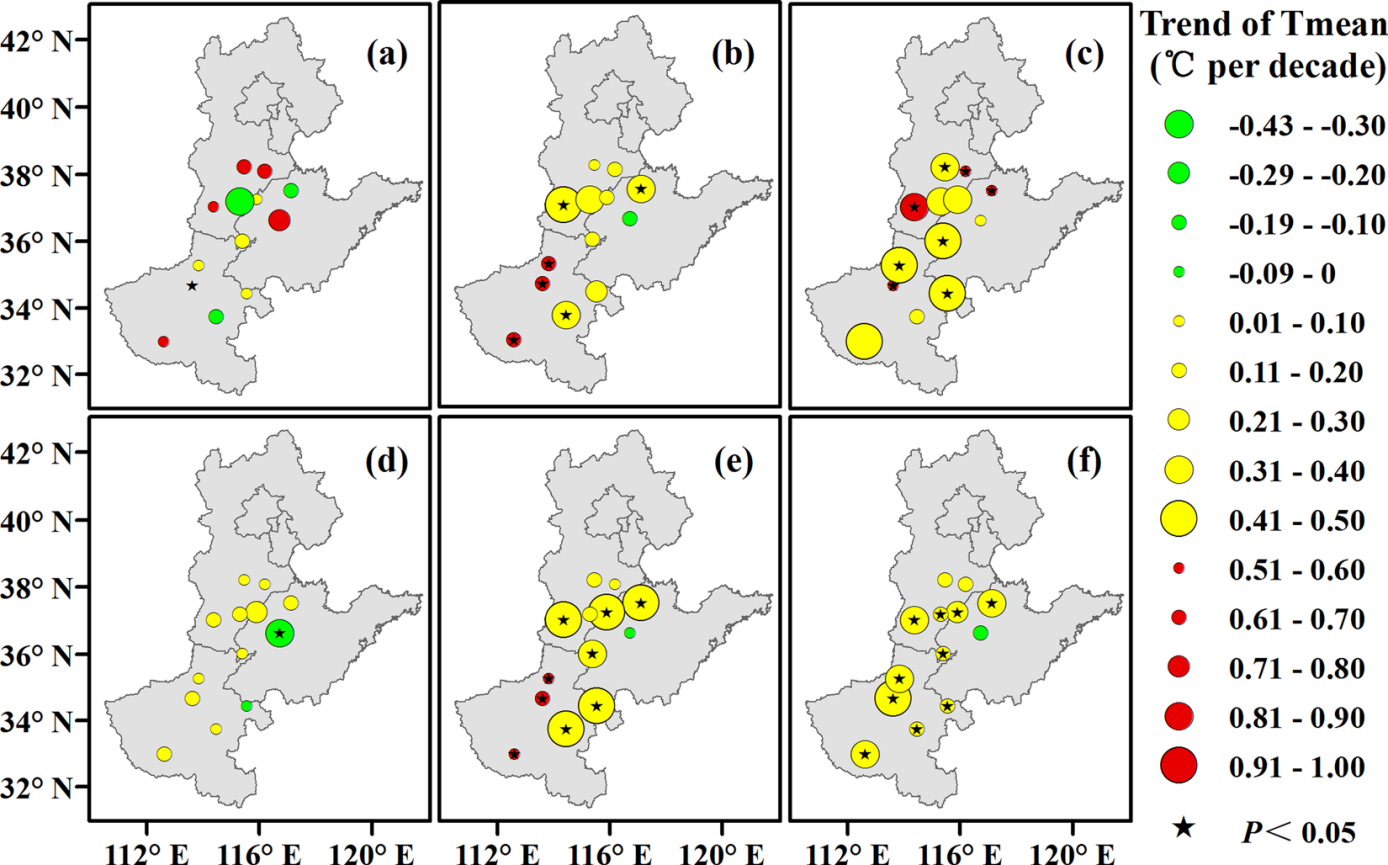
Trends in Tmean during the sowing-emergence (**a**), emergence-squaring (**b**), squaring-flowering (**c**), flowering-boll opening (**d**), and boll opening-harvest (**e**) periods and the whole growing period (**f**) of cotton across the North China Plain from 1981 to 2012. Green, yellow, and red circles represent the trends of the Tmean. Black pentagrams represent a significance level of 0.05. Maps were generated using ArcGIS 10.1 (ESRI Inc, Redlands, CA, USA, http://www.esri.com/).

On average, a general warming trend was observed (Fig. 4). The Tmean values during the sowing-emergence, emergence-squaring, squaring-flowering, flowering-boll opening, and boll opening-harvest periods and the whole growing period increased 0.29, 0.31, 0.43, 0.05, 0.36, and 0.24 °C per decade on average, respectively. From 1981 to 2012, the Tmean values during the sowing-emergence, emergence-squaring, squaring-flowering, flowering-boll opening, and boll opening-harvest periods and the whole growing period increased at 10 (77.0%), 12 (92.3%), 13 (100.0%), 11 (84.6%),12 (92.3%) and 12 (92.3%) stations, respectively, and this increase was significant at 1 (7.7%), 6 (46.2%), 8 (61.5%), 0 (0%), 9 (69.2%) and 10 (77.0%) stations, respectively, although decreases were also observed at several stations.

**Figure 5:**
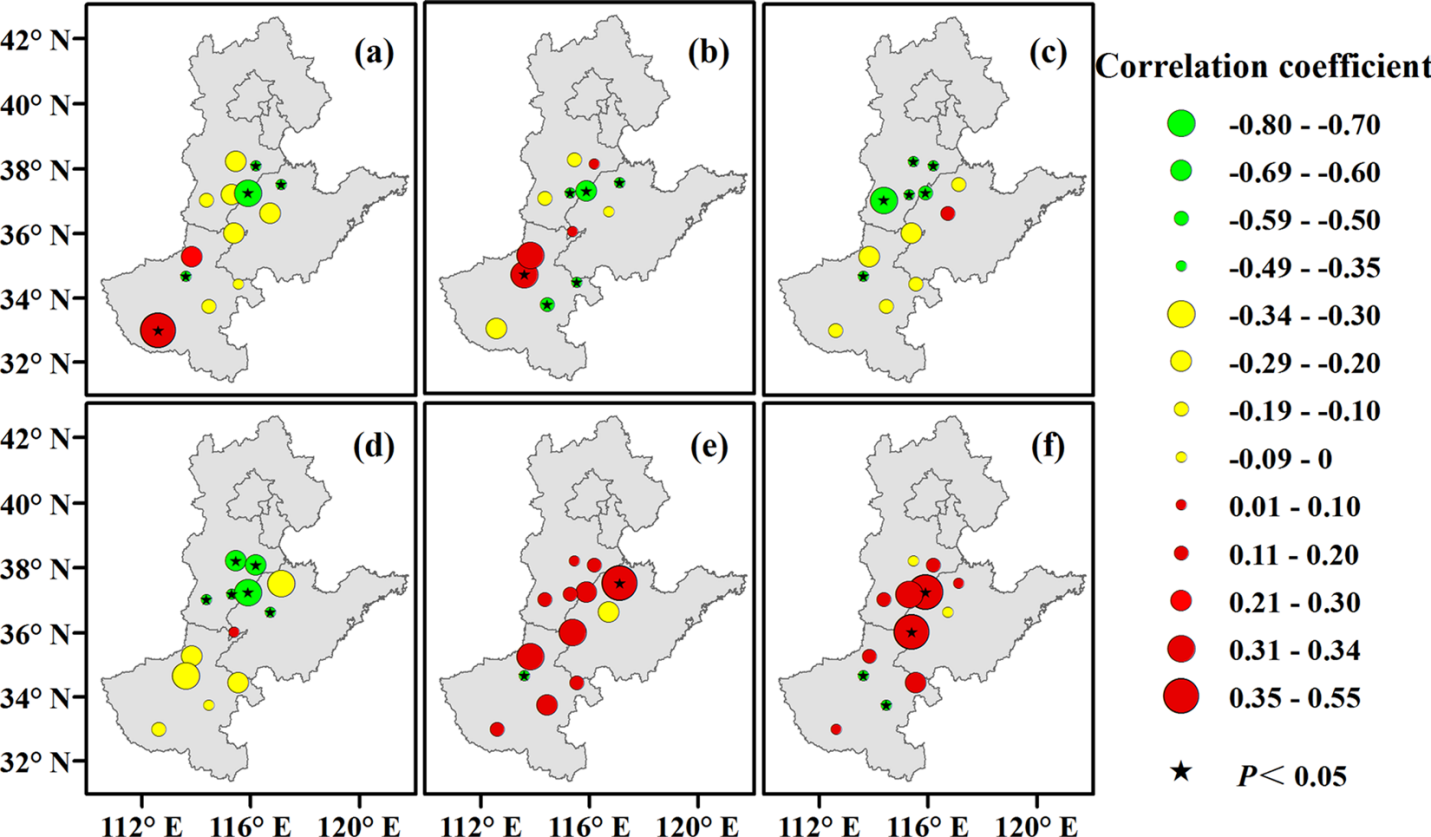
Correlation between Tmean and the lengths of the sowing-emergence (**a**), emergence-squaring (**b**), squaring-flowering (**c**), flowering-boll opening (**d**), and boll opening-harvest (**e**) periods and the whole growing period (**f**) of cotton across the North China Plain from 1981 to 2012. Green, yellow, and red circles represent the correlation coefficient. Black pentagrams represent a significance level of 0.05. Maps were generated using ArcGIS 10.1 (ESRI Inc, Redlands, CA, USA, http://www.esri.com/).

The correlation coefficients between Tmean and the lengths of the sowing-emergence, emergence-squaring, squaring-flowering and flowering-boll opening periods were generally negative, at −0.20, −0.17, −0.29, −0.35 on average, respectively, while the correlation coefficients between Tmean and the boll opening-harvest and whole growing periods were generally positive, at 0.20 and 0.11 on average, respectively (Fig. 1c). A negative correlation was found at 11 (84.6%), 9 (69.2%), 12 (92.3%), and 12 (92.3%) stations, and this correlation was significant at 4 (30.8%), 5 (38.5%), 6 (46.2%), and 6 (46.2%) stations, respectively (*P* < 0.05). In contrast, the correlation coefficients between Tmean and the lengths of the boll opening-harvest period and the whole growing period were positive at 11 (84.6%) and 10 (77.0%) stations, respectively. These correlations were significant at 1 (7.7%) and 2 (15.4%) stations, respectively (*P* < 0.05) (Fig. 5).

### Relationship between cotton phenology and rainfall

**Figure 6:**
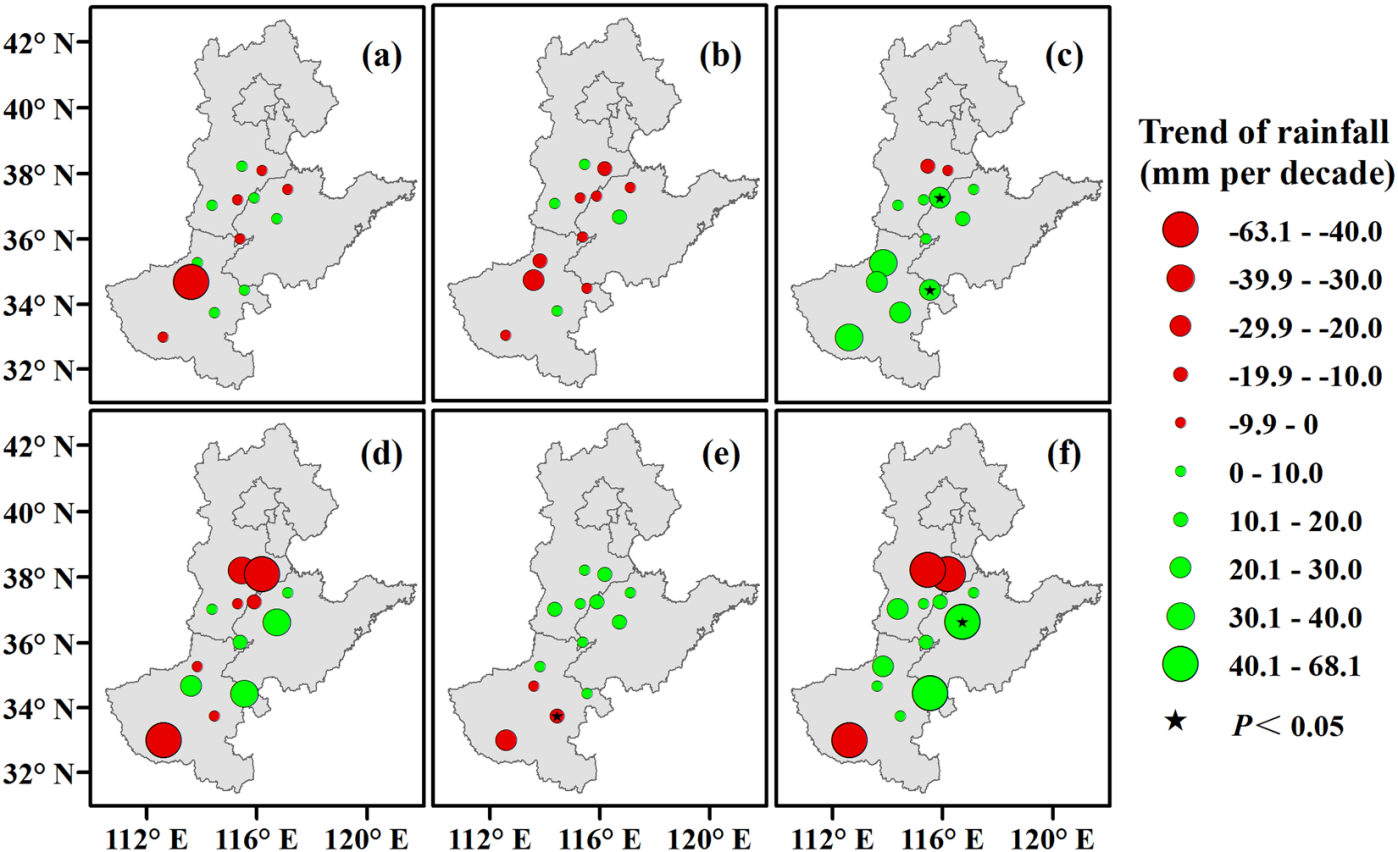
Rainfall trends during the sowing-emergence (**a**), emergence-squaring (**b**), squaring-flowering (**c**), flowering-boll opening (**d**), and boll opening-harvest (**e**) periods and the whole growing period (**f**) of cotton across the North China Plain from 1981 to 2012. Green and red circles represent the trends of rainfall. Black pentagrams represent a significance level of 0.05. Maps were generated using ArcGIS 10.1 (ESRI Inc, Redlands, CA, USA, http://www.esri.com/).

The rainfall values during the squaring-flowering and boll opening-harvest periods and the whole growing period were increased by 14.08, 1.69, and 6.85 mm per decade on average, respectively (Fig. 6). An increasing rainfall trend was found at 11 (84.6%), 10 (76.9%), and 10 (76.9%) stations during the squaring-flowering and boll opening-harvest periods and the whole growing period, respectively.

**Figure 7:**
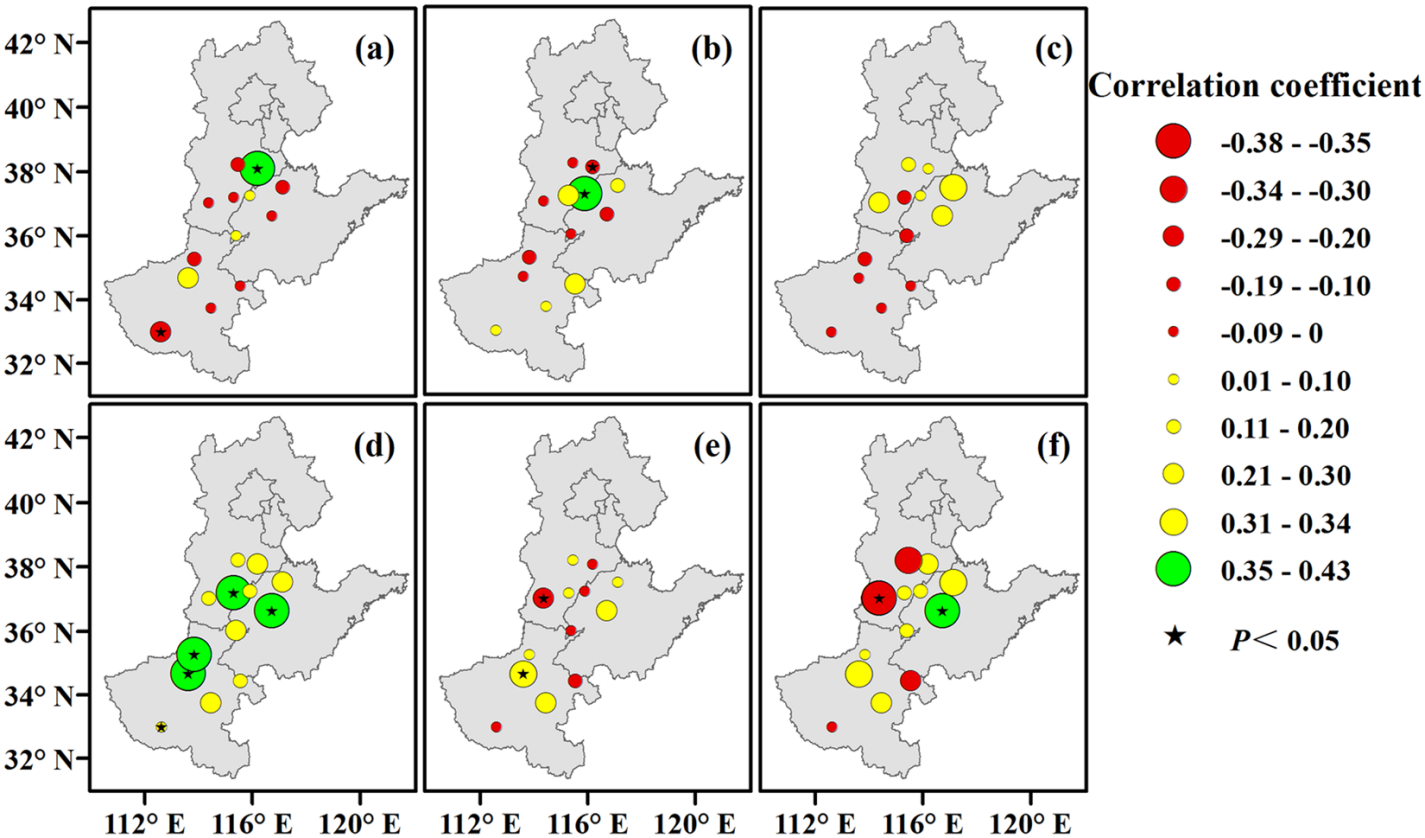
Correlation between rainfall and the lengths of the sowing-emergence (**a**), emergence-squaring (**b**), squaring-flowering (**c**), flowering-boll opening (**d**), and boll opening-harvest (**e**) periods and the whole growing period (**f**) of cotton across the North China Plain from 1981 to 2012. Green, yellow, and red circles represent the correlation coefficient. Black pentagrams represent a significance level of 0.05. Maps were generated using ArcGIS 10.1 (ESRI Inc, Redlands, CA, USA, http://www.esri.com/).

The correlation coefficients between rainfall and the lengths of the sowing-emergence were generally negative, at 0.05 on average, while the correlation coefficients between rainfall and the length of the flowering-boll opening periods and the whole growing period were generally positive, at 0.23 and 0.10 on average, respectively (Fig. 1d). The length of the sowing-emergence period was negatively correlated with rainfall at 9 (69.2%) stations (Fig. 7). Furthermore, the correlation coefficient between rainfall and the length of the flowering-boll opening and the whole growing periods was positive at 11 (84.6%) and 9 (69.2%) stations, respectively.

## Discussion

### Response of cotton phenology to changes in temperature and rainfall

Many recent studies have documented an explicit change in crop phenology across broad regions due to climate change^22–24^. However, most studies have focused on major food crops, and only a few have addressed trends of cotton phenology in the context of climate change^8^. The present study assessed trends in cotton phenology, Tmean and rainfall on the North China Plain with respect to different cotton growing periods from 1981 to 2012. Furthermore, the relationships of the length of cotton growing periods with Tmean and rainfall were investigated. The results showed that the sowing, emergence, squaring, flowering, and boll opening dates of cotton were advanced by 0.24, 1.29, 0.91, 2.71 and 0.82 days per decade on average, respectively, but the cotton harvest date was delayed by 0.28 days on average per decade. Furthermore, while the lengths of sowing-emergence, emergence-squaring, and squaring-flowering periods decreased, those of the flowering-boll opening and boll opening-harvest periods increased by 1.28 and 2.20 days per decade on average, respectively.

Many studies have shown that crop growth may be accelerated by warming. The length of the wheat, barley, and rapeseed growing periods has decreased in the last several decades^13, 16, 25^. In the present study, warming trends occurred during different growing periods. In addition, the lengths of the sowing-emergence, emergence-squaring, squaring-flowering, and the flowering-boll opening periods were negatively correlated with Tmean. The results were generally consistent with previous research on other crops^13, 16^. However, the lengths of the boll opening-harvest and whole growing periods were positively correlated with Tmean; climate warming lengthened the boll opening-harvest period by 5.58 day per °C on average. Thus, except for the delayed harvest date, all other cotton phenological dates were advanced, which indicated that cotton growth was accelerated by warming. However, the harvest date was delayed because of the indeterminate growth habit of cotton^26^, generally resulting in shorter growing periods before flowering but longer growing periods after flowering. Ultimately, the whole growing period was generally shorter in the southern part but longer in the northern part of the North China Plain.

Warming significantly impacts crop phenology^9, 15^. However, few studies have investigated the impact of changes in rainfall on cotton phenology. In the present study, increasing trends in rainfall were noted at 11 (84.6%), 10 (76.9%), and 10 (76.9%) stations for the squaring-flowering and boll opening-harvest periods and the whole growing period, respectively. Furthermore, the lengths of the squaring-flowering and boll opening-harvest periods and the whole growing period were positively correlated with rainfall. Therefore, changes in rainfall would prolong the lengths of the squaring-flowering and boll opening-harvest periods and the whole growing period. However, the whole growing period of cotton in the southern part of the North China Plain decreased, which may be due to the impact of the change in Tmean on cotton phenology.

### Comparisons with previous studies in other crops and other regions

Previous studies showed a delay in the sowing date of winter wheat of 1.5 days per decade on average but an advance in the harvest date of 1.4 days per decade; therefore, the whole growing period was shortened^15^. Wang *et al*.^27^ reported that the summer maize sowing date occurred relatively early at 13 (48.1%) of 27 stations examined on the North China Plain, while maturity dates were significantly delayed at 10 (37.0%) stations; thus, the length of the whole growing period increased at 18 (63.0%) stations. Comparisons between these two crops showed that winter wheat and summer maize exhibited opposite results, whereas the results for cotton were more complex. These differences may be due to an impact of soil temperature on the sowing date and a warming trend in recent decades that has caused delayed sowing of winter wheat and advanced sowing of summer maize; however, because plastic film mulching technology has been applied for cotton, climate warming has less effect on cotton sowing date. Furthermore, the maturity date of winter wheat may have occurred early because warming may have accelerated crop growth and shortened the growing period. The maturity date of summer maize has been delayed because farmers have adopted appropriate farming practices and cultivars in response to climate warming, such as double delay technology for winter wheat and summer maize^27^. The harvest date of cotton has generally been advanced in the southern part of the North China Plain and delayed in the northern part, which may due to the indeterminate growth habit of cotton and the fact that the southern portion exhibits greater accumulated temperatures. Nevertheless, Lu *et al*. reported that increased temperatures advanced cotton phenology but whole growth period lengths were prolonged or shortened in various stations of eastern Australia, which was generally consistent with this study^28^. However, Huang and Ji reported that climate warming prolonged the whole growth period length of cotton in Xinjiang Province of China^8^.

Previous studies about other crops conducted in Europe and on the U.S. Great Plains have shown strong negative links between temperature and phenology, and the agricultural phases of many crops have been advanced due to increased temperatures^22, 23, 29, 30^. These findings are similar to our results. However, the lengths of the boll opening-harvest period and the whole growing period were positively correlated with Tmean in the present study, possibly because of the indeterminate growth habit of cotton. The indeterminate growth habit of cotton means that as long as the environment is suitable for cotton, it could continue to grow^26^. Furthermore, the majority of published studies have investigated the relationship between temperature change and crop phenology, but only a few studies have investigated the relationship between changes in rainfall and crop phenology^29^. The results of the present study showed that the relationship between rainfall and the length of the whole growing period was positive.

### Countermeasures for the impact of climate change on cotton phenology

Considering the increasing trend of temperature in relation to the cotton sowing time and the indeterminate growth habit of cotton, adjusting the sowing date would be beneficial for rational utilization of the heat resource to extend the period of cotton growth and to develop and improve the cotton yield potential. Moreover, farmers could make full use of the higher temperature during cotton sowing by adopting deep planting and other supporting technology to avoid the use of plastic film mulching during sowing and reduce “White Pollution”. Furthermore, this study indicated that flowering occurred earlier as a result of climate change. The risk of heat damage could increase by a combination of warming, the change in the date of flowering, and the particular sensitivity of cotton to extreme heat during flowering. Therefore, heat-resistant varieties and appropriate cultivation practices are required to reduce the impact of excessive heat. In addition, this study indicated that a warming trend in recent decades has delayed the cotton harvest date in the northern part of the North China Plain; therefore, adopting cultivars with a longer growing period would benefit future cotton production in the northern part of the North China Plain. Nevertheless, the temperature is higher after boll opening, and changes in farming practices, such as adjusting the sowing date and the use of newer cultivars, will therefore be necessary to adapt to climate warming.

### Potential limitations of this study

This study investigated the spatiotemporal changes in cotton phenology and the relationships between cotton phenology and climate change. However, the impact of climate change on cotton phenology was not quantified. Changes in cotton phenology were found to occur in response to the combined effects of temperature, rainfall, agronomic management practices, and cultivars. Therefore, the conclusions that can be drawn from this study are limited. Addressing these limitations will require further structured experimental approaches, such as factorial experiments or model simulations. Additionally, the effect of climate change on cotton yields was not considered in this study and is a topic that we will address in the future.

## Conclusions

This study investigated the spatiotemporal changes in cotton phenology and the length of different cotton growing phases and explored the relationship between the lengths of different cotton growing phases and climate change on the North China Plain from 1981 to 2012. The results showed that the sowing, emergence, squaring, flowering, and boll opening dates generally advanced, but harvest was delayed in the northern part and advanced in the southern part of the North China Plain. The sowing-emergence, emergence-squaring, and squaring-flowering periods of cotton were shortened by 1.50, 1.94, and 1.96 days per decade on average, respectively, but the flowering-boll opening and boll opening-harvest periods were prolonged on average by 1.28 and 2.20 days per decade, respectively. The lengths of the sowing-emergence, emergence-squaring, squaring-flowering, and flowering-boll opening periods were negatively correlated with Tmean, whereas the lengths of the boll opening-harvest period and the whole growing period were positively correlated with Tmean. The results indicated that the phenology of cotton on the North China Plain has been significantly impacted by climate change and that such changes in cotton phenology affect the development and yield of cotton. Therefore, adjusting the sowing date, using heat-resistant varieties, and adopting appropriate cultivation practices will be necessary to combat climate change in the future. These results support the exploration of appropriate agricultural strategies and policies to adapt to global climate change on the North China Plain and in other areas with a similar ecology.

## Materials and Methods

### Study region

**Figure 8:**
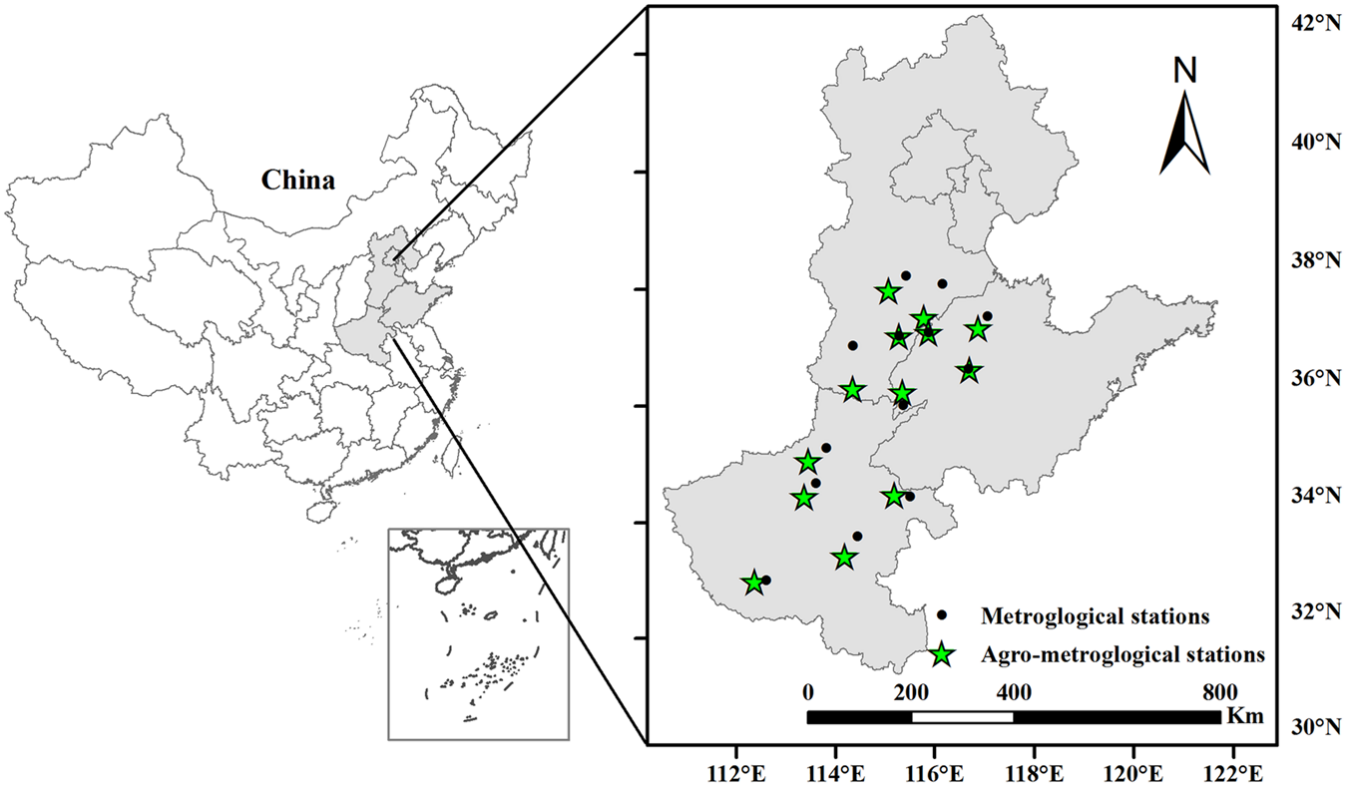
Locations and spatial distribution of the metrological and agro-meteorological stations on the North China Plain. A map of China showing the locations of the three provinces and municipalities within the country (shaded area). Black dots and green pentagrams represent the meteorological and agro-meteorological stations, respectively. Lines indicate province boundaries. Maps were generated using ArcGIS 10.1 (ESRI Inc, Redlands, CA, USA, http://www.esri.com/).

The North China Plain extends from 112° to 122°E longitude and 32° to 42°N latitude, covering three provinces (Shandong, Hebei, and Henan) and two municipalities (Beijing and Tianjin; Fig. 8). Cotton is the largest cash crop by area in this region. This region exhibits average annual production of 2.07 million tons of cotton, accounting for 30.89% of China’s total cotton production from 2005 to 2014^20^. Therefore, in the context of climate change, investigating cotton phenology on the North China Plain is important for the stability of cotton production in China.

### Historical cotton phenology and climate data

Cotton phenological data from 1981 to 2012 were obtained from 13 agro-meteorological experimental stations maintained by the China Meteorological Administration. The Julian dates (day of year; DOY) of six phenological stages (sowing, emergence, squaring, flowering, boll opening, and harvest) were recorded at each station. A standardized observation method was used to collect the phenological data. On alternate days, observations were recorded by well-trained agricultural technicians. Each phenological event was clearly defined. For example, the squaring date was recorded when 50% of the cotton was budding, and the boll opening date was recorded when 50% of the bolls on the cotton plants were open^31^. The whole growing period from sowing to maturity was divided into five phases, which included sowing-emergence, emergence-squaring, squaring-flowering, flowering-boll opening, and boll opening-harvest^31^.

Meteorological observation data, including the daily maximum and minimum temperatures and precipitation, were also collected from the China Meteorological Administration for the same period. Three of the agro-meteorological stations were located at the same sites as the meteorological stations, and the remaining 10 agro-meteorological stations were located near the meteorological stations (Fig. 8). Using the interpolated daily temperature data, the daily mean temperature was estimated as the average of the daily minimum and maximum air temperatures for each agrometeorological station.

### Data analysis

Trends of cotton phenology, length of growing periods, mean temperatures, and rainfall were calculated using Microsoft Office 2013 software (Redmond, WA, USA). The statistical significance of any trends was tested using a two-tailed t test in the SPSS 11.0 analytical software package (SPSS Inc., Chicago, IL, USA). Correlations of the lengths of the different growing phases with mean temperature (Tmean) or rainfall were investigated using Pearson’s correlation analysis. The spatial distributions of phenology and climate data were displayed using ArcGIS 10.1 software (ESRI Inc, Redlands, CA, USA, http://www.esri.com/).
